# Three types of dermal grafts in rats: the importance of mechanical property and structural design

**DOI:** 10.1186/1475-925X-12-125

**Published:** 2013-12-04

**Authors:** Chuangang You, Xingang Wang, Yurong Zheng, Chunmao Han

**Affiliations:** 1 Department of Burn, the Second Affiliated Hospital of Zhejiang University, Hangzhou 310009, China

**Keywords:** Knitted mesh, Microstructure, Angiogenesis, Tissue regeneration, Mechanical properties

## Abstract

**Background:**

To determine how the mechanical property and micro structure affect tissue regeneration and angiogenesis, three types of scaffolds were studied. Acellular dermal matrices (ADM), produced from human skin by removing the epidermis and cells, has been widely used in wound healing because of its high mechanical strength. Collagen scaffolds (CS) incorporated with poly(glycolide-co-L-lactide) (PLGA) mesh forms a well-supported hybrid dermal equivalent poly(glycolide-co-L-lactide) mesh/collagen scaffolds (PMCS). We designed this scaffold to enhance the CS mechanical property. These three different dermal substitutes—ADM, CS and PMCSs are different in the tensile properties and microstructure.

**Methods:**

Several basic physical characteristics of dermal substitutes were investigated in vitro. To characterize the angiogenesis and tissue regeneration, the materials were embedded subcutaneously in Sprague–Dawley (SD) rats. At weeks 1, 2, 4 and 8 post-surgery, the tissue specimens were harvested for histology, immunohistochemistry and real-time quantitative PCR (RT-qPCR).

**Results:**

In vitro studies demonstrated ADM had a higher Young’s modulus (6.94 MPa) rather than CS (0.19 MPa) and PMCS (3.33 MPa) groups in the wet state. Compared with ADMs and CSs, PMCSs with three-dimensional porous structures resembling skin and moderate mechanical properties can promote tissue ingrowth more quickly after implantation. In addition, the vascularization of the PMCS group is more obvious than that of the other two groups. The incorporation of a PLGA knitted mesh in CSs can improve the mechanical properties with little influence on the three-dimensional porous microstructure. After implantation, PMCSs can resist the contraction and promote cell infiltration, neotissue formation and blood vessel ingrowth, especially from the mesh side. Although ADM has high mechanical strength, its vascularization is poor because the pore size is too small. In conclusion, the mechanical properties of scaffolds are important for maintaining the three-dimensional microarchitecture of constructs used to induce tissue regeneration and vascularization.

**Conclusion:**

The results illustrated that tissue regeneration requires the proper pore size and an appropriate mechanical property like PMCS which could satisfy these conditions to sustain growth.

## Background

Full-thickness skin defects resulting from injury, burns and nonhealing ulcers represent a significant clinical problem that is far from being solved. At present, the main treatments for skin defects are transplantations such as autografts, allografts and xenografts to ensure wound closure as soon as possible [1,2]. Tissue engineering of whole skin represents an equally attractive and ambitious novel approach [3]. Many skin tissue engineering methods have been developed for the treatment of full-thickness skin defects [4,5]. Although tissue engineering has a significant potential to provide alternative approaches for skin regeneration, several problems have hampered progress in translating technological advances to clinical reality [6,7]. The skin split-thickness autograft has been widely used but frequently leads to scarring in full-thickness wounds because of the lack of dermis. Therefore, dermal reconstruction or regeneration is the critical issue to reduce scar formation and improve the quality of wound healing; this has encouraged the initiation and development of dermal substitutes [8-12]. Several products have been manufactured and used clinically. However, none of them were able to perfectly regenerate skin for various reasons, including unsuitable physical or mechanical properties and inappropriate microstructures that hinder the cellular infiltration, proliferation and differentiation [13]. The ideal substitutes should have suitable properties that effectively induce rapid angiogenesis, a significant challenge in the field of tissue engineering and regenerative medicine, which is one of the crucial premises to promote regeneration and decrease the risk of infection [14,15].

Dermal regeneration scaffolds regulate the tissue and cells’ regeneration as an external force during the process of extension and growth of cells in the material. These materials are capable of not only inducing tissue regeneration and vascularization but can also inhibit the hyperplasia. Studies have proven that collagen-based scaffolds possess many interesting properties but also that they possess weak mechanical properties for skin tissue engineering applications [16-18]. Many approaches, such as physical/chemical crosslinking, have been developed to improve the biomechanical function of scaffolds, and the results seem obvious [19-21]. Some researchers have reported that a knitted mesh from artificial polymers is a good way to provide collagen-based scaffolds with excellent mechanical properties, and this type of hybrid scaffold has been successfully applied to repair cartilage, ligaments, tendons, blood vessels, etc. [22-26]. In our previous study, we found that mesh-reinforced collagen-chitosan scaffolds can resist contraction and promote cell infiltration, neotissue formation, and blood vessel ingrowth more effectively than collagen-chitosan scaffolds [27]. Based on these studies, we developed poly(L-lactide-co-glycolide) (PLGA) yarns knitted into mesh-reinforced collagen scaffolds to enhance the collagen scaffolds’ mechanical properties. For skin tissue engineering, several types of meshes knitted by PLGA or poly(lactic acid-co-caprolactone) (PLACL) were integrated with collagen to fabricate dermal substitutes, and the primary results illustrated that using the knitted mesh as a “skeleton” improved the mechanical strength of the hybrid scaffolds and inhibited wound contraction [27-29].

ADM matrix, which is rich in predominantly type-I collagen, is increasingly used in reconstructive surgery applications [30]. Therefore, the in vivo influence of this type of hybrid scaffold on inductive regeneration and angiogenesis has been widely investigated in skin tissue engineering. Compared with artificial polymers, ADM materials (ADMs) as naturally derived biomaterials have excellent mechanical properties, but the pores are too small to sustain the vascularization and facilitate cell infiltration [31].

Collagen scaffold, PLGA-Collagen scaffold and ADM are three types of dermal substitutes composed of collagen with different mechanical properties and microstructure characteristics. What type of role does the biomechanics and microstructure play in wound healing? In this study, we chose these three types of scaffolds mentioned above to represent the low (CS), intermediate (PMCS), and high (ADM) mechanical property scaffolds to determine which one is most suitable for dermal regeneration. PMCS was prepared by incorporating the PLGA knitted mesh (PLGAm) into collagen scaffold (CS) as a hybrid scaffold to study the function of the mechanical properties and microstructure in tissue regeneration. Young’s modulus as a physical quantity of the ability to resist deformation of solid material was measured to illustrate the mechanical properties. The hypothesis that tissue regeneration and angiogenesis is possibly dependent on porous microstructure and suitable mechanical properties of scaffolds was tested by investigating the angiogenic and regenerative potentials of the three scaffolds implanted subcutaneously in a rat model. The study protocol was approved by Institutional Review Board at the Zhejiang University.

## Materials and methods

### Materials

PLGA (LA/GA = 1:9, molecular weight: 80–110 kD) yarns were provided by Foryou Medical Devices Co., LTD (China), and Genipin was purchased from Linchuan Zhixin biological technology Co., LTD (China). Type I collagen was extracted and purified from fresh bovine tendons by swelling, deleting telopeptides with pepsin, and sufficient dialysis as described previously [14,27]. ADM, purchased from Beijing Jayyalife biological Technology Co., Ltd. is native dermal matrix from cadaver skin by removing the epidermis and other cells contain a complete basement membrane. It is milky white and flexible which could be used as “permanent” dermal substitute materials. Analytical-graded reagents and deionized water were utilized in the experiments.

### Manufacturing of PLGA mesh

The PLGA yarns (25 filaments/yarn; diameter of filament, 15 μm) were knitted into a network with a braiding machine (HKS3-M, China). The knitted fabrics were rinsed in acetone for 15 min, washed in deionized water with ultrasonic waves for 15 min × 3 times, desiccated in the vacuum dryer at 45°C for 24 h, and then thermally formed at 105°C for 24 h. Finally, they were stored under vacuum at 4°C prior to application.

### One-step crosslinking to prepare CS and PMCS

The PMCS was prepared by integrating knitted mesh into porous CS. First, bovine type Ι collagen was dissolved in 0.5 M acetic acid and a 0.5% genipin mixed solution to form a 0.05% (w/v) solution [32,33]. In our previous work, we compared different crosslink concentration of genipin, the suitable concentration of genipin was determined by analysis of pore size, mechanical strength and other activities. The PLGA mesh was cut into designated sizes (4.0 cm × 4.0 cm) and then extended on the flat plate of the homemade model (4.0 cm × 4.0 cm × 0.2 cm). A 3.2 ml collagen/chitosan solution was poured in carefully, and the mesh was kept unfolded. The composite was standing at −4°C for 24 h, frozen at −25°C for 3 h, and then lyophilized for 16 h to produce the hybrid scaffold. The porous PMCS was prepared and treated as previously described [27,34].

Before being implanted in animal experiments, the scaffolds were sterilized with 70% ethanol for 30 min and then washed completely with sterilized phosphate buffer solution (PBS) 5 times, each for 15 min.

### Physical evaluation

After being sectioned with a razor blade and coated with platinum, the morphologies of ADM, CS and PMCS were observed using SEM (Philips XL30, Eindhoven, The Netherlands). The accelerating voltage was set at 1.0 kV. To determine the mean pore size of the scaffolds, images of cross-sectioned surfaces from CS and PMCS were analyzed as described previously [27]. Three SEM images of each scaffold were taken randomly. In each, ten apparent pores were selected and measured with a ruler. The long and minor axes of pores in a perpendicular direction were measured, and the average was taken as the mean pore size.

Young’s modulus of ADM, CS and PMCS in dry and wet conditions was measured with a universal testing machine (Instron, model 5543, High Wycombe, UK). Before the tests, the samples were trimmed into 4.0 cm × 1.0 cm pieces. Six samples in one group were divided into two subgroups. One subgroup was prepared for the dry measurement, and the other was first immersed in PBS for 1 h and then measured. Each sample was stretched at a rate of 1 mm/min at 20 ± 2°C. Finally, the ultimate Young’s modulus was calculated according to formula 1 [35].

$$\mathrm{EM}=\left(F/A0\right)/\left(\mathrm{\Delta L}/L0\right)$$

Formula 1. EM is the elasticity modulus; F is the mechanical force applied to the sample; A0 is the original cross-sectioned area; ΔL is the change amount by which the length of sample; and L0 is the original length of the sample.

### In vivo implantation

All the animal experimental procedures were carried out under the Zhejiang University animal care and use committee. Adult Sprague–Dawley (SD) rats, male, aged 2 months and weighing 200 ± 8 g, were purchased from the Experimental Animal Centre of Zhejiang University. After the hair on the backs of the rats was trimmed and removed, the rats were anesthetized by an intraperitoneal injection of 3% pentobarbital sodium solution (Sigma) at a dosage of 1.0 mL/kg. After local disinfection with 2.5% povidone iodine solution, the sterilized ADM, PMCS and CS scaffolds (diameter 2.0 cm) were embedded into the subcutaneous tissue pockets of rats. At intervals of 1, 2, 4, and 8 weeks after the operation, the rats were killed with a lethal dose of pentobarbital sodium solution, and then the rat skin was incised and turned open along the midline on the back to expose the embedded objects. Pictures of the implants were taken, and the tissue specimens were obtained and kept in 10% neutral formalin aqueous solution and liquid nitrogen for histopathological observation and molecular biological detection, respectively. At each time point, six parallel scaffolds were set for each group.

### Histology

The tissue samples were prepared and embedded in paraffin. The paraffin blocks were all sectioned at a thickness of 5 μm and stained by hematoxylin & eosin (HE) staining and Masson trichrome staining. The stained sections were visualized under an optical microscope.

### Immunohistochemistry

The infiltration of blood vessels into the implants was evaluated morphologically by examining the critical factors relative to the process of angiogenesis such as CD31 and α-SMA by immunohistochemistry.

For the immunohistochemical staining, the pretreatment of paraffin-embedded tissue samples was conducted as described before [36]. After heat repairing of antigens in 0.01 M citrate buffer aqueous solution at 95°C for 6 ~ 8 min, the nonspecific antigens were blocked with 5% goat serum at 37°C for 30 min. Then, the sections were exposed to rabbit anti-CD31 primary antibody (1:100, Abcam, Cambridge, UK) at 4°C overnight. After 3 washes with PBS, the sections were incubated with goat anti-rabbit secondary antibody at 37°C for 30 min, developed and counterstained with 3, 3’-diaminobenzidine tetrahydrochloride (DAB) solution and hematoxylin, respectively. The positive areas stained with brown color were observed under an optical microscope.

### RNA isolation and real-time semiquantitative PCR analysis

The scaffolds embedded subcutaneously in the rats for 1, 2, 4, and 8 weeks were harvested to isolate RNA for the RT-sPCR analysis. Each sample was dissolved in 1 mL of Trizol reagent (Invitrogen, CA, USA), and RNA was isolated. The content and purity of RNA were measured with an ultraviolet spectrophotometer after dissolving RNA in DEPC-H_2_O solution. RNA conversion to cDNA was carried out with an M-MLV Reverse Transcriptase cDNA synthesis kit (Promega, WI, USA). RT-sPCR was amplified for CD31, VEGF, PDGF-BB and α-SMA and the calibrator reference gene (glyceraldehyde-3-phosphate dehydrogenase, GAPDH). The reaction was performed with iQ^TM^5 Multiple Real-time Fluorescent Quantitation PCR Determinator (Bio-Rad, USA) for 40 cycles. The expression level of each target gene was normalized to GAPDH and calculated using Bio-Rad icycler software (version 2.0). The fluorescent primers were devised with Primer Express 2.0 (ABI, USA) and Beacon Designer software (Bio-Rad, USA) and were obtained from Shanghai Bioengineering Co., Ltd. (China). The detailed sequences are shown in Table 1.

**Table 1:** Real-time primer sequences and condition

**Gene**	**Source**^**a**^	**Primer sequences**^**b**^	**Size(bp)**	**Annealing**
CD31	NM031591.1	F 5-GGAGGTGACAGAAGGTGGGATTG-3	66	60°C
		R 5-GCTTGGCAGCGAAACACTAACAGG-3		
PDGFBB	NM031524	F 5-CCCACACGTCAAACTACAGCTCCAA-3	112	60°C
		R 5-GCCCAGTTCGTTTCAGTGCCACAT-3		
α-SMA	NM031004	F 5-CCAGGGAGTGATGGTTGGAATG-3	98	60°C
		R 5-GCCGTGTTCTATCGGATACTTCAG-3		
VEGF	NM031836	F 5-GGCTTTACTGCTGTACCTCCACCAT-3	161	60°C
		R 5-CGGGGTACTCCTGGAAGATGTC-3		
GAPDH	NM017008	F 5-GGTGGACCTCATGGCCTACAT-3	88	60°C
		R 5-GCCTCTCTCTTGCTCTCAGTATCCT-3		

^a^Indicates GeneBank accession numbers where applicable. ^b^Indicates the sequences of forward (F) and reverse (R) primers.

Ration, respectively (n = 3).

### Statistical analysis

All quantitative data were analyzed with SPSS 16.0 (USA) and presented as the mean ± standard deviation. Significant differences among specimens were evaluated by a *t*-test. A value of p < 0.05 was regarded as significant.

## Results

### Physical observation

**Figure 1:**
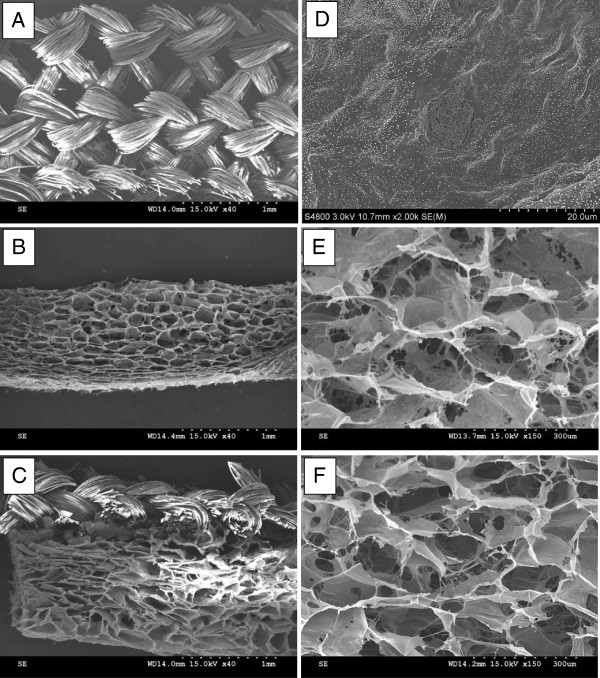
SEM images PLGA mesh (A), CS (B, E), PMCS (C, F) and ADM (D).

The microstructures of ADM, CS and PMCS are shown in Figure 1. The PLGAm had a net-like structure with a loop size of 350 ± 12 μm (Figure 1A). CS had a mean pore size of 118 ± 5 μm and a porosity of 94% measured by ethanol inhalation (Figure 1B, E); the pores were not obvious in ADM (Figure 1B) [37]. In the hybrid scaffold, the mesh was located near the lower side and interconnected contiguously with collagen/chitosan structures (Figure 1C). Meanwhile, the collagen/chitosan sponge in the PMCS remained porous and interconnected and occupied the openings of the PLGA mesh (Figure 1C, F). The collagen/chitosan sponge had an average pore size of 112 ± 5 μm and a porosity of 91%, similar to those of CS.

**Figure 2:**
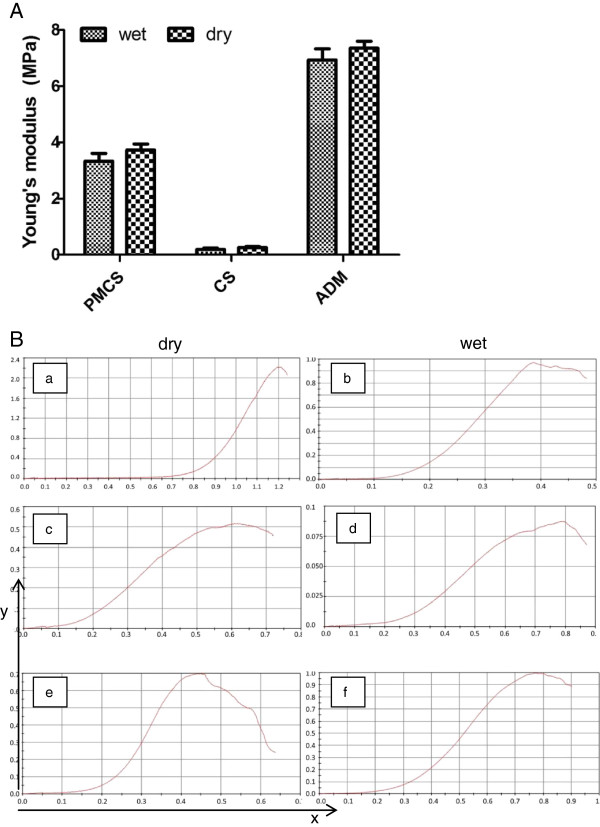
**Young’s modulus of PMCS, CS and ADM. (A) **Young’s modulus of PMCS, CS and ADM, respectively (n = 6), p < 0.001. **(B)** The representative stress–strain curves for the three scaffolds in both dry and wet state: **(a, b)** ADM, **(c, d)** CS, **(e, f)** PMCS. The X-axis represented tensile strain (mm/mm) and the Y-axis represented tensile stress (MPa). The calculation was completed by the computer. Generally, the slope of the curve is the Young’s modulus.

Figure 2 illustrates the Young’s modulus of ADM, CS and PMCS in wet and dry states. In wet state, the Young’ modulus of CS was much lower (0.19 MPa) than the others. Meanwhile, PMCS retained suitable Young’s modulus (3.33 MPa) similar to that of human native dermis (1.03-3.10 MPa) [38]. While ADM was the highest (6.94 MPa).

### Gross observation

For all 3 groups, no evident inflammatory reaction, e.g., infection, fistula and fibrous capsule, was observed in the implantation and adjacent sites at different time points. At week 1, the implants had integrated into the surrounding tissues, and several blood vessels could be observed around the implants in PMCS and CS. This process was not obvious in ADM. ADM and PMCS maintained a similar shape and area with the first state. Meanwhile, the residual area of CS reached nearly 70% of the previous area.

Two weeks after implantation, more blood vessels grew into the implants and the color of the implants turned to light red in the PMCS and CS groups. Several blood vessels could be observed around the ADM group, but the surrounding tissue was not close. In the PMCS group, the mesh loops became indistinguishable, which possibly resulted from the neotissue ingrowth induced by the scaffolds. The residual areas of ADM and PMCS changed little, while CS underwent obvious contraction.

From weeks 2 to 4, more abundant blood vessels could be visualized in and around the PMCS and CS groups compared with ADM, and the outlines became indistinct. The appearances of scaffolds were similar to the normal tissue. The residual areas of PMCS and ADM were significantly larger than those of CS. With the increase of implantation time, most of the scaffold structures gradually became invisible. At week 8, all the implants were assimilated completely and invisible on gross examination.

### Histology

**Figure 3:**
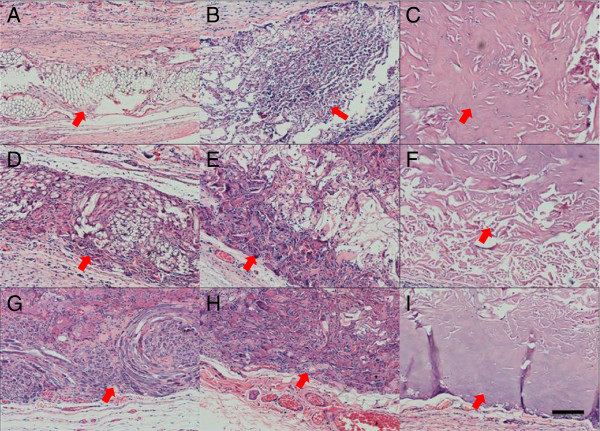
**HE staining of PMCS, CS and ADM.** HE staining of sections of the PMCS **(A, D, G)**, CS **(B, E, H)** and AD **(C, F, I)** implanted for 1, 2, 3 weeks after operation. The Bar indicates 100 μm. The red arrows indicate the scaffolds.

The implants were harvested at different times for histological analysis to evaluate the tissue response to the three different types of scaffolds, and the results of HE staining are shown in Figure 3. At week 1, the implants were easily distinguished from the host tissues in all the groups. Inflammatory cells such as granulocytes and macrophages were observed at this stage. In the PMCS group, fibroblasts grew into the implants from the mesh side rapidly, and abundant ECM deposition was observed in the center of PMCS (Figures 3A, D, G). In the ADM and CS groups, the infiltration of fibroblasts was mainly distributed around the scaffold with a thin ECM secretion (Figures 3A, B). Additionally, newly formed blood vessels in the PMCS were observed, while most areas in the center of the CS and ADM did not exhibit cell or capillary infiltration (Figures 3A, B, C). Two weeks after implantation, the implants were closely integrated with the adjacent tissues, except in the ADM group. The number of granulocytes decreased, and the number of macrophages increased in all three groups. The three-dimensional structures of PMCS were almost filled by cells, ECM and newly formed capillaries (Figures 3D, G), which was not clear in the other two groups. Meanwhile, blank areas without cell ingrowth remained in the CS and ADM groups (Figures 3H, I). At week 4, newly formed tissue became more abundant, and the number of macrophages showed down-regulation in all the groups. The degradation of the three types of scaffolds was more obvious than before, and their outlines were difficult to discriminate from the host tissues. Especially in the PMCS group, the newly formed tissue integrated well with the surrounding tissues without an obvious gap (Figures 3D, G). From weeks 4 to 8, CS and PMCS degraded completely, while some residual components remained in the ADM group (Figures 3G-I).

### Masson trichrome staining

**Figure 4:**
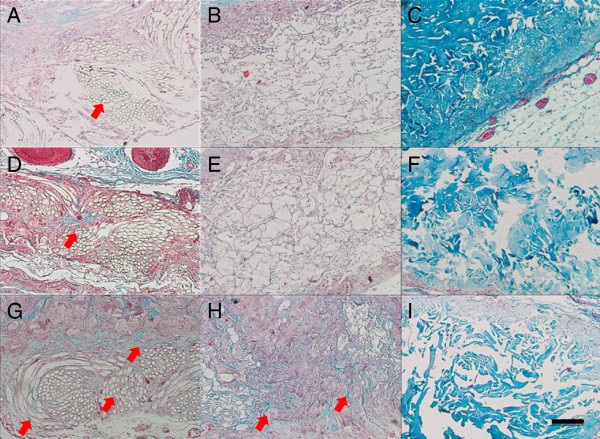
**Masson staining of sections of the PMCS, CS and AD.** Masson staining of sections of the PMCS **(A, D, G)**, CS **(B, E, H)** and AD **(C, F, I)** implanted for 1, 2, 3 weeks after operation. The Bar indicates 100 μm. Light blue indicates newly-formed collagen, and red indicates cytoplasma.

To investigate collagen deposition, Masson trichrome staining was performed, and the results are illustrated in Figure 4. In all the groups, the quantity of collagen deposition increased with the prolongation of the implantation time. At week 1, many blue collagen fibrils were observed near to the mesh side in the PMCS group, while a few fibrils were distributed around the ADM and CS groups. At week 2, compared with the collagen deposition throughout the PMCS, there were some fibrils positioned in the scaffolds near to the surfaces in the other groups. At week 4, more collagen fibrils appeared in the PMCS group, and the fibrils were more homogenous and ordered than those of other groups. From weeks 4 to 8, more abundant and ordered collagen deposition was observed in the PMCS group, while the CS and ADM groups revealed a heterogeneous collagen distribution.

### Immunohistochemistry

**Figure 5:**
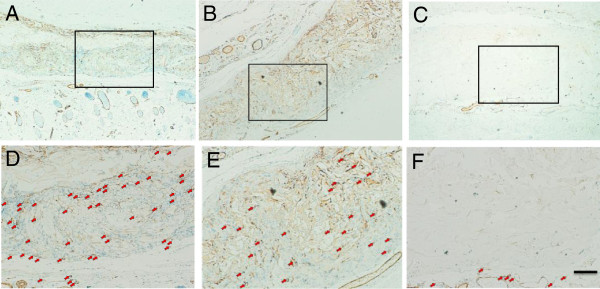
**α-sma immunohistochenmical staining.** α-sma immunohistochenmical staining of sections of the PMCS **(A, D)**, CS **(B, E)** and AD **(C, F)** for 2 weeks after operation. **D**, **E**, **F** are the enlargement of the bos area in **A**, **B**, **C**. The Bar indicates 100 μm. Arrows indicate blood vessels.

**Figure 6:**
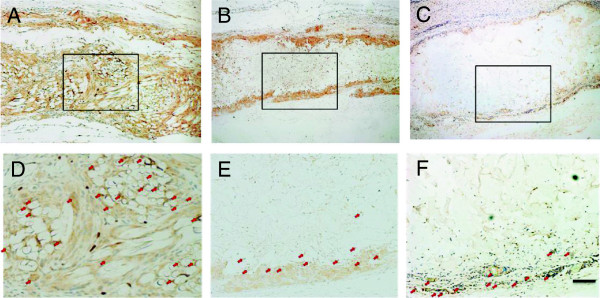
**CD31 immunohistochenmical staining.** CD31 immunohistochenmical staining of sections of the PMCS **(A, D)**, CS **(B, E)** and AD **(C, F)** for 2 weeks after operation. The Bar indicates 100 μm. Arrows indicate blood vessels.

To characterize the newly formed blood vessels in the implants, α-SMA, CD31 (a marker of endothelial cells around the blood vessels) immunohistochemical staining was performed, and the results are illustrated in Figure 5 and Figure 6. This indicates that the number of blood vessels increased to some extent for all the groups with the development of the implantation time, and their expression levels were influenced heavily by the type of implant. In week 1, the blood vessels were chiefly distributed around the implants, particularly in the PMCS group, and the density was significantly higher than that in CS and ADM. At week 2, the blood vessel densities of PMCS increased, respectively, and the values are also significantly higher than those of CS and ADM. At 4 and 8 weeks after implantation, the PMCS and CS groups displayed strong positive staining, while some blank fields could be found in the ADM group. The number of blood vessels in the PMCS group continued to increase and was higher than that of other groups with a significant difference at the different time points.

### RT-qPCR analysis

**Figure 7:**
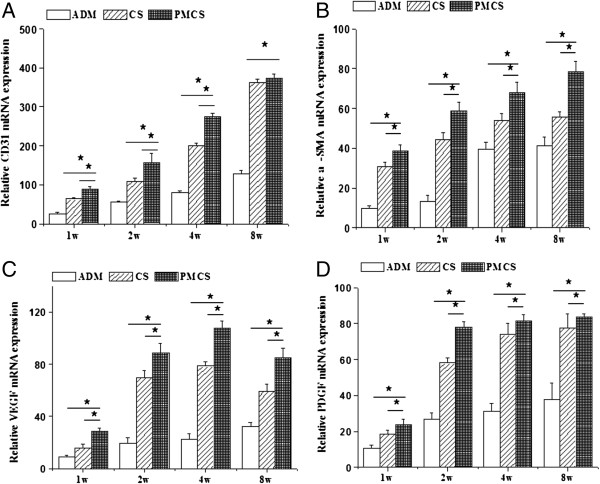
**Real-time quantitative analysis of CD31, α-SMA, VEGF and PDGF-BB.** Real-time quantitative analysis of CD31 **(A)**, α-SMA **(B)**, VEGF **(C)** and PDGF-BB **(D)** mRNA expression of the implants at 1, 2, 4 weeks after operation, respectively (n = 3). * indicates statistically significant difference, p < 0.05.

Angiogenesis at the gene level was further characterized by quantifying several critical factors, such as CD31, α-SMA, VEGF and PDGF-BB, using RT-qPCR. Generally, the trends of gene expression in the 3 groups fluctuate regardless of the types of genes, whereas the PMCS group always retained a higher gene expression level at different time points. When compared within the different implantation periods, the CD31, α-SMA, VEGF and PDGF-BB mRNA expressions of the PMCS group were always significantly higher than those of other groups (Figure 7).

## Discussion

The biology mechanical property participates in the whole process of wound healing. Activated and proliferating fibroblasts derived from local resting cells induced by tensile stress and contract the wound matrix through a stress fiber–rich contractile apparatus appear in the wound 2–3 d after injury. In response to tensile stress, proto-myofibroblasts differentiate into highly contractile myofibroblasts that express α-SMA [39]. Biomechanical stress generated by fibroblasts that migrate into the fibrin matrix from tissues surrounding the wound or wound contraction could direct translocation of the vasculature and regulate the other cells’ biological behavior [40,41]. Interconnected by gap junctions, myofibroblasts secrete extracellular matrix components and at the same time contract the wound by transmitting tension across intracellular actin stress fibers connected to the extra-cellular matrix [40]. Engineered scaffolds just replace the role of extra-cellular matrix which should possess such a mechanical and structural pattern for angiogenesis.

Tissue engineering is based on the idea of biomimetic materials science to emulate the target tissue or organ closely in terms of material composition, structure, material surface properties and mechanical properties and to devise a substitute [42,43]. Natural tissue engineering materials include the main component of the extracellular matrix from the animal body, and some other organisms extract. Cell aggregation and tissue formation induced by scaffolds must be supplied with enough nutrients by the vascular system, indicating that the velocity of vascular infiltration should match that of tissue formation. The rapid construction of microcirculation in the equivalents can facilitate cell adhesion, proliferation and functionalization by transmitting oxygen and nutrients and taking metabolic waste away. Therefore modification of the scaffolds’ physicochemical properties is important to enhance the function. The biological environment provided by biomimetic scaffolds is a network of structural and functional components [44,45]. Many researchers have studied the relationship between mechanical strength and dermal regeneration. Techniques such as optimizing the pore size to enhance its mechanical property indicated that three-dimensional porous structures commonly characterized by porosity, pore size and interconnectivity can support cell migration and guide vascular infiltration [27,34,46]. Some in vitro studies focusing on the optimal mean pore size of collagen-based scaffolds indicated that large pores (≥250 μm) favor cell attachment, proliferation and migration [47-51]. In addition to crosslinking which could improve the microstructure and mechanical properties are the most basic methods of biomimetic scaffolds. It is obvious that the biomechanical strength plays an important role in wound healing, but what type of mechanical property and microstructure a perfect dermal scaffolds should have is still unsolved. In this research we compared three different dermal scaffolds to explore the reasonable mechanical property and microstructure.

CS and AMD are model dermal substitutes that are designed based on the wide application of collagen scaffolds. Collagen scaffolding has been proven to possess excellent biocompatibility and biodegradability and is a quite suitable material for skin tissue engineering [52,53]. However, without sufficient mechanical properties, it is always compressed or crumpled and cannot maintain its porous structure. It was demonstrated by the test of Young’s modulus (Figure 2). The knitted mesh in this experiment is composed of PLGA (LA/GA = 1:9) yarns, which are polyesters and have been approved by the FDA for clinical use. In a previous study, the PLGA mesh was incorporated into collagen scaffolds to assemble PMCSs and support structures for 3D porous structures (Figure 1B, E) for cell aggregation and vascular infiltration. The introduction of PLGA mesh into CS had little influence on the microstructure of the collagen sponge (Figure 1D, E) and improved the mechanical properties of the hybrid scaffold to a level similar to that of normal dermis (1.03-3.10 MPa) (Figure 2) [38].

ADM is prepared from human dermal tissue by removing the cells and epidermis. ADM has been widely used for wound regeneration because of its excellent biocompatibility and mechanical properties that mimic human acellular dermis through its ability to naturally interface with host tissues with minimal tissue response [54,55]. However, its high mechanical property is based on a small and collapsed aperture which could be observed on Figure 1C.

The angiogenesis of all three groups was systematically investigated. Gross observation provides direct evidence of angiogenesis at the early implantation stage. As the implantation time and vessel density increased, advanced testing methods were needed to count the number of the newly formed blood vessels. In this study, newly formed blood vessels were specially marked by immunochemical staining for CD31 (Figure 6), a type I integral membrane glycoprotein expressed by endothelial cells early in vascular formation [56]. With the maturation of the blood vessels, another cell layer composed of smooth muscle cells occurred around the endothelial layer, and this layer expresses α-SMA (Figure 5). Compared with CS, the knitted mesh mechanically supports the 3D porous microstructure of the collagen sponge, facilitating the transmission of oxygen and nutrients and the infiltration of cells and blood vessels. In addition, the proper mechanical strength can also change regularly according to the requirement of neotissue and new blood vessel growth. Although the mechanical properties of ADM are better than those of PMCS, the angiogenesis results are not good. The histology result showed that the infiltration of fibroblasts and vessels was mainly distributed around the scaffolds (Figure 3). The microstructures of ADM showed the collapsed hole and tousle structure with burliness fiberboard tier upon tier. The high mechanical strength of ADM made it relatively difficult to deform, which blocked the tissue regeneration. Further evidence of angiogenesis was provided by RT-qPCR, which was used to investigate the expressions of CD31, α-SMA, VEGF and PDGF-BB at the gene and protein levels (Figure 7). Among these key factor related to vascular development, VEGF can promote vascular development and maturation with a tiny dose; PDGF-BB, often released by endothelia cells at the sprouting tip of forming capillaries, is not involved in the initial vessel formation but in the neovessel stability and functionalization by inducing anastomoses and mediating pericyte recruitment [57,58]. The results of RT-qPCR indicate that the PMCS group exhibited the highest mRNA expression of VEGF and PDGF-BB, gradually increasing from weeks 1 to 4 and decreasing in week 8. The results of molecular biological detections for CD31 and α-SMA also demonstrated that the PMCS group had a much higher level of angiogenesis than the other two groups. All these results confirm that moderate mechanical properties and proper physical structure have a basic and critical influence on the rapid angiogenesis of dermal equivalents. Only the proper mechanical strength and aperture could promote the tissue and cell ingrowth. Both CS and ADM have homogeneous holes and compositions, but the PMCS has a variable structure with a mesh positioned at one end that produces a 3D mode with a graded aperture in which the holes from the mesh side to the collagen side are gradually enlarged. This is in line with the general laws of the material-induced regeneration of tissue and cells.

Tissue engineering has proven to be one of the most promising alternative therapies for wound healing and tissue regeneration [59]. In this study, scaffold degradation and in situ tissue regeneration can be observed simultaneously. In the in vivo microenvironment, the degradation of scaffolds is a complex process involving hydrolyzation and biodegradation. CS required no less than 4 weeks to be assimilated by the host tissue, in contrast to ADM and PMCS [14,53]. In the PMCS group, the outlines of PLGA fibers remained relatively intact at weeks 1 and 2, displayed obvious degradation phenomena with fiber fracturing and crushing, and almost degraded completely with a little residue surrounded by macrophages and neotissue at week 8, which resulted in a stronger resistance to contraction in the early stage than that of CS (Figure 3A, D, G). Almost 8 weeks later, the outline of the ADM was still visible with HE (Figure 3C, F, I) and Masson’s staining (Figure 4C, F, I). Fibroblast infiltration and collagen secretion in the 3 types of implants were visualized well by HE and Masson’s staining. From week 1 to 2, the PMCS group promoted the fastest cell infiltration and ECM formation, especially in the portion near the mesh side, which resulted from the rapid angiogenesis described above. Abundant and ordered collagen deposition was closely related to the up-regulation of fibroblast function in the local microenvironment. It has been reported that cell activity is mainly regulated by the specific surface area (SSA) in the scaffold, from which the space distribution is determined by the geometrical structure of the constructs [48]. In the in vivo complex biomechanical environment, the existing knitted mesh in the PMCS can maintain a geometrical microstructure and SSA distribution suitable for fibroblast activity to reach a higher level of tissue regeneration induced by the hybrid scaffold. Scaffolds should possess suitable mechanical properties to maintain 3D porous structures for tissue ingrowth and provide temporary mechanical support until the regenerated tissue can support mechanical loads.

## Conclusions

The rational selection and collocation of biomaterials help design biomimetic DRT. Incorporation of the PLGA knitted mesh into a collagen/chitosan porous scaffold with lyophilization formed a reinforced hybrid scaffold with excellent mechanical property and a suitable microstructure. When embedded into the dorsal subcutaneous pockets, PMCS can resist the contraction, induce cell infiltration and neo-tissue formation, and promote blood vessel ingrowth compared with CS. ADM has a high mechanical property that is large enough to resist contraction, but without a porous structure and elastic strain capacity, the angiogenesis that induces tissue regeneration is delayed. Hence, the ideal scaffolds not only require the proper mechanical strength to maintain their structure but also require an elasticity change to accommodate the extension of neo-tissue. This indicates that suitable mechanical functioning could be one of the most crucial characteristics for maintaining the shape and microstructure of the constructs to facilitate cell ingrowth and functionalization. In summary, the mechanical properties and microstructure, which play an important role in accelerating angiogenesis and inducing in situ tissue regeneration, are the most basic characteristics of engineered scaffolds.

## Abbreviations

ADM: Acellular dermal matrices; CS: Collagen scaffolds; PLGA: Poly(glycolide-co-L-lactide); PMCS: Poly(glycolide-co-L-lactide) mesh/collagen scaffolds; DAB: 3, 3′-diaminobenzidine tetrahydrochloride; GAPDH: Glyceraldehyde-3-phosphate dehydrogenase.

## Competing interests

The authors declare that they have no competing interests.

## Authors’ contributions

CGY performed experiments and analysis and wrote the paper. XGW performed experiments and analysis. YRZ carried out pathology experiment. CMH conceived of the study, and participated in its design and helped to draft the manuscript. All authors read and approved the final manuscript.
